# Targeting POLE2 Creates a Novel Vulnerability in Renal Cell Carcinoma via Modulating Stanniocalcin 1

**DOI:** 10.3389/fcell.2021.622344

**Published:** 2021-02-11

**Authors:** Chuanjie Zhang, Yan Shen, Lili Gao, Xiaojing Wang, Da Huang, Xin Xie, Danfeng Xu, Hongchao He

**Affiliations:** ^1^Department of Urology, Shanghai Ruijin Hospital Affiliated to Shanghai Jiao Tong University School of Medicine, Shanghai, China; ^2^Research Center for Experimental Medicine, Shanghai Ruijin Hospital Affiliated to Shanghai Jiao Tong University School of Medicine, Shanghai, China; ^3^Department of Pathology, Shanghai Ruijin Hospital Affiliated to Shanghai Jiao Tong University School of Medicine, Shanghai, China

**Keywords:** POLE2, renal cell carcinoma, proliferation, migration, stanniocalcin 1

## Abstract

**Objective:**

The aim of this study is to investigate the biological functions and the underlying mechanisms of DNA polymerase epsilon subunit 2 (POLE2) in renal cell carcinoma (RCC).

**Methods:**

The datasets of POLE2 expression in The Cancer Genome Atlas Kidney Clear Cell Carcinoma (TCGA-KIRC) and International Cancer Genome Consortium (ICGC) databases was selected and the correlation between POLE2 and various clinicopathological parameters was analyzed. The POLE2 expression in RCC tissues was examined by immunohistochemistry. The POLE2 knockdown cell lines were constructed. *In vitro* and *in vivo* experiments were carried out to investigate the function of POLE2 on cellular biology of RCC, including cell viability assay, clone formation assay, flow cytometry, wound-healing assay, Transwell assay, qRT-PCR, Western blot, etc. Besides, microarray, co-immunoprecipitation, rescue experiment, and Western blot were used to investigate the molecular mechanisms underlying the functions of POLE2.

**Results:**

POLE2 was overexpressed in RCC tissues, and high expression of POLE2 was correlated with poor prognosis of RCC. Furthermore, knockdown of POLE2 significantly inhibited cell proliferation, migration, and facilitated apoptosis *in vitro*. *In vivo* experiments revealed that POLE2 attenuated RCC tumorigenesis and tumor growth. we also illuminated that stanniocalcin 1 (STC1) was a downstream gene of POLE2, which promoted the occurrence and development of RCC. Besides, knockdown of POLE2 significantly upregulated the expression levels of Bad and p21 while the expression levels of HSP70, IGF-I, IGF-II, survivin, and sTNF-R1 were significantly downregulated. Western blot analysis also showed that knockdown of POLE2 inhibited the expression levels of Cancer-related pathway proteins including p-Akt, CCND1, MAPK9, and PIK3CA.

**Conclusion:**

Knockdown of POLE2 attenuates RCC cells proliferation and migration by regulating STC1, suggesting that POLE2-STC1 may become a potential target for RCC therapy.

## Introduction

Renal cell carcinoma (RCC) is one of the most common malignant tumors of the urinary system, with a higher morbidity and mortality, second only to bladder cancer in urinary system tumors (Chen et al., 2016; Bray et al., 2018). Totally 25 to 30% of RCC patients have metastasized at the time of diagnosis (Zhao et al., 2014). Although radical nephrectomy is an effective method for the treatment of early and locally advanced RCC, about 30% of patients have metastases after surgery (Jiang et al., 2008). Patients with metastatic kidney cancer still have a poor prognosis and limited treatment options. Therefore, finding novel treatment strategies or prognostic biomarkers of RCC is of great significance for improving the prognosis of RCC.

DNA polymerase epsilon subunit 2 (POLE2) is a subunit of DNA polymerases localized in the nucleus, which commonly present in DNA repair (Su et al., 2020). Currently, POLE2 have reported to be abnormally expressed in breast cancer, colorectal cancer, mantle cell lymphoma, bladder cancer and lung adenocarcinoma (Hartmann et al., 2008; Zhou et al., 2008; Zekri et al., 2015; Chubb et al., 2016; Li et al., 2018). Besides, Wu et al. (2020) have found that high expression of POLE2 is a biomarker for poor prognosis in squamous cell lung cancer. However, few studies have reported the relationship between POLE2 and RCC and its regulatory mechanism (Su et al., 2020). Therefore, in this study, we comprehensively investigated the POLE2 expression and its role and mechanism in RCC from the biological, cellular and animal levels, then clarified that POLE2 was a tumor-promoting gene of RCC, and high POLE2 expression indicated poor prognosis.

## Materials and Methods

### Patient Samples

The RNA-seq datasets and clinical information of RCC patients were downloaded from The Cancer Genome Atlas (TCGA)^1^ and International Cancer Genome Consortium (ICGC)^2^ databases. The 83 pairs of RCC tissues and corresponding adjacent normal tissues were obtained from RCC patients undergoing partial or radical nephrectomy from July 2012 to February 2014 at Shanghai Ruijin Hospital. The clinical information was obtained from the patients’ medical records, including age, gender, tumor size, grade, stage, and pathological T. The RCC specimens and adjacent normal tissues were placed in liquid nitrogen for storage. Besides, there biopsies were collected from three RCC patients guided by the computed tomography (CT) and the patient derived organoids (PDOs) were established and cultured as previously described (Vlachogiannis et al., 2018).

This study was approved by the Ethics Committee of Shanghai Ruijin Hospital, and all patients have provided written informed consents.

### Cell Lines and Culture

The human RCC cell lines (A498 and ACHN) were purchased from Cell Bank, Shanghai Institute of Biochemistry and Cell Biology, Chinese Academy of Sciences (Shanghai, China). All cells were maintained in Roswell Park Memorial Institute (RPMI)-1640 medium (Gibco, Scotland, United Kingdom) containing 10% fetal bovine serum (Sigma, St. Louis, MO, United States), 100 U/ml penicillin, and 100 μg/ml streptomycin. All cell lines were incubated at 37°C with 5% CO_2_.

### Lentiviral Vector Construction and Cell Infection

The short hairpin RNA (shRNA) to knock down expression of POLE2 or stanniocalcin 1 (STC1) in A498 and ACHN cells were designed using the following sequences: 5′-TTCT CCGAACGTGTCACGT-3′ (shCtrl), 5′-CGATTGTTCTTGG AATGATA-3′ (POLE2-shRNA), 5′-CGTGAAGACTTAGTAA ATAA-3′ (POLE2#2-shRNA), 5′-TAAATTTGACACTCAGGG AAA-3′ (STC1-shRNA). The synthesized DNA oligonucleotides were annealed to form double-stranded DNA and inserted into the *Age*I/*Eco*RI sites of linearized vector BR-V-108 carrying a green fluorescent protein (GFP) gene (Shanghai Yibeirui Biomedical Technology Co., Ltd). After amplification and DNA sequence confirmation, these lenti-shRNA vectors were co-transfected into 293T cells with two packaging plasmids pHelper 1.0 and pHelper 2.0 (Shanghai Yibeirui Biomedical Technology Co., Ltd) for 48 h. Lentiviral particles were purified from the culture supernatant. A498 and ACHN cells were infected in 6-well plates at the density of 2 × 10^5^ cells/well at a multiplicity of infection (MOI) of 10. Infection efficiency was evaluated under a florescence microscope. The POLE2 knockdown efficiency was calculated by quantitative reverse transcriptase-polymerase chain reaction (qRT-PCR) and western blot, respectively.

### qRT-PCR

Total RNA was isolated by using TRIzol reagent (cat. no. T9424-100m; Sigma-Aldrich), and cDNA was synthesized using the Hiscript QRT supermix for qPCR (+gDNA WIPER) (cat. no. R123-01; Vazyme Biotech Co., Ltd) according to the manufacturer’s instructions. Then qRT-PCR was performed using Real time PCR instrument (cat. no. VII7; ABI company) with the AceQ qPCR SYBR Green master mix (cat. no. R111-02; Vazyme Biotech Co., Ltd). The PCR condition was as following: 95°C for 60 s, followed by 45 cycles of 95°C for 10 s, and 60°C for 30 s. The sequences of primers were used as shown in Supplementary Table 1.

### Western Blot

Total proteins were extracted by 1x lysis buffer and protein concentration was determined using the BCA Protein Assay Kit (cat. no. 23225; HyClone-Pierce). Then 20 μg protein samples were loaded to 10% sodium dodecyl sulfate-polyacrylamide gel electrophoresis (SDS-PAGE), followed by a transfer onto polyvinylidene difluoride (PVDF) membranes. After blocking with 5% skim milk-Tris-based saline-Tween 20 (TBST) at room temperature for 1 h, membranes were incubated overnight at 4°C with primary antibodies to POLE2 (dilution at 1:1000; cat. no. ab180214; Abcam), N-cadherin (dilution at 1:1000; cat. no. ab18203; Abcam), Vimentin (dilution at 1:1000; cat. no. ab92547; Abcam), Snail (dilution at 1:1000; cat. no. ab194583; CST), BCL-2 (dilution at 1:1000; cat. no. 3879S; Abcam), CDC42EP3 (dilution at 1:1000; cat. no. NBP1-88382; NOVUS), EZR (dilution at 1:2000; cat. no. ab40839; abcam), GDAP1 (dilution at 1:1000; cat. no. orb39831; biorbyt), RDM1 (dilution at 1:1000; cat. no. orb352658; biorbyt), and STC1 (dilution at 1:1000; cat. no. ab229477; abcam), GAPDH (dilution at 1:3000; cat. no. AP0063; Bioworld) served as loading control. After washed with TBST three times, membranes were then incubated with the matching goat anti-rabbit (dilution at 1:3,000; cat. no. A0208; Beyotime) for 2 h at room temperature. Proteins were visualized using an ECL + plus^TM^ Western Blotting system kit (cat. no. RPN2232; Amersham), and then scanned and analyzed by ImageJ.

### Cell Viability Assay

Lentivirus-infected A498 and ACHN cells in the logarithmic phase were reseeded into 96-well plates (cat. no. 3599; Corning Inc) at a density of 2,000 cells/well. After cultured for 1, 2, 3, 4, and 5 days, cells were incubated with 20 μl MTT (3-[4,5-dimethylthiazol-2-yl]-2,5-diphenyltetrazolium bromide) (5 mg/ml) for 4 h and the optical density (OD) at 490 nm was measured by a microplate reader (cat. no. M2009PR; Tecan Infinite) according to the manufacturer’s instructions.

### Clone Formation Assay

Cells in the logarithmic phase were reseeded into 6-well plates at a density of 500 cells/well and incubated for 8 days. The growth medium of each well was refreshed every 3 days. At the end of each experiment, the cells were fixed with 1 ml 4% paraformaldehyde for 30–60 min, then washed with phosphate-buffered saline (PBS) and stained with 500 μl Giemsa (AR-0752; Shanghai Dingguo Biotechnology Co., Ltd) for 10–20 min. Cell colonies were photographed under a microscope and cell colonies were counted.

### Soft Agar Colony Formation Assay

A 2 ml gel base medium containing 10% FBS and 0.7% agar was used for soft agar colony assay. On the basis of this, cells at the density of 1 × 10^5^ cells/well were seeded in 2 ml of medium containing 10% FBS with 0.35% agar and incubated at 37°C for 21 days. The photographs of colonies developed in soft agar were taken using Olympus IX5 microscope, and the number of colonies was scored by ImageJ software (NIH, United States).

### Transwell Assay

Cells in the logarithmic phase (8 × 10^4^ cells/well) were resuspended in serum-free medium and placed in the upper chamber of the Transwell, while 600 μl medium containing 30% FBS was added to the lower chamber. After 24 h, cells remaining on the upper surface of the chamber were removed by cotton swabs. Migrated cells were stained with 0.1% crystal violet, and counted under a microscope (IX73; Olympus).

### Wound Healing Assay

Cells were seeded into 96-well plates a density of 50,000 cells/well and cultured at 37°C with 5% CO_2_. When cells grew more than 90% confluence, a scratch was made using a scratch tester aligned the center of the lower end of the 96-well plate. The cells were washed with PBS twice and cultured in 0.5% PBS with 5% CO_2_ at 37°C. Photograph were captured by Cellomics (ArrayScan VT1, Thermo) at the time point (0 and 24 h) and analyzed the migration area with Cellomics.

### Apoptosis Assay

Cells in the logarithmic phase were washed and resuspended at 1 × 10^6^ cells/ml, and then proceeded with eBioscience^TM^ Annexin V Apoptosis Detection Kit APC (cat. no. 88-8007-74; eBioscience) as per manufacturer’s instructions. The cells were analyzed by flow cytometry (Guava easyCyte HT; Millipore).

### Co-immunoprecipitation (Co-IP)

All steps of co-immunoprecipitation were performed at 4°C. The ACHN cells were washed with pre-cooled PBS twice and treated with the lysis buffer for 5–10 min. After centrifuged at 13,000 × *g* for 10 min, the total protein was collected and the concentration was examined by the BCA Protein Assay Kit. The selected groups of protein were added to the corresponding centrifuge tubes, and added with 1 μg rabbit IgG or 1 μg corresponding immunoprecipitating antibodies, then incubated overnight.

Each tube was added with 20 μl Protein A/G PLUS-Agarose beads and incubated for 1–2 h, then the tube was centrifugated at 2,000 × *g* for 1 min and the supernatant was removed. The Protein A/G Plus-Agarose beads were rinsed by 1 ml lysis buffer and centrifugated at 2,000 × *g* for 1 min, followed by the removal of supernatants, which was repeated twice. Then the Protein A/G Plus-Agarose beads were added with 5x loading buffer, boiled for 5–10 min. The supernatant was collected and transferred to a new tube for Co-immunoprecipitation with the antibody against DYKDDDDK Tag (dilution at 1:1000; cat. no. 14793; CST) POLE2 (dilution at 1:1000; cat. no. ab180214; abcam), STC1 (dilution at 1:1000; cat. no. ab124891; abcam) and GAPDH. The specific test procedure was similar to Western blot analysis.

### Animal Xenografts Study

A total of 20 BALB/c nude mice (female, 4-week-old) were obtained from Shanghai Lingchang Biotechnology Co., Ltd [animal production license number: SCXK (Shanghai) 2018-0003]. The mice had *ad libitum* access to a pellet diet and water and were maintained in well-ventilated rooms with a controlled environment of 12 h light/dark cycle and temperature of 28 ± 2°C. All animal experiments were approved by the Ethics Committee of Shanghai Ruijin Hospital.

The mice were randomly divided into shCtrl and shPOLE2 groups (*n* = 10). ACHN cells (4 × 10^6^ cells/mouse), stably transfected with shCtrl or shPOLE2, were subcutaneously injected into the right armpit of the mice. After 2 weeks, the body weights and tumor volumes were monitored 1–2 times a week. At the end of the study (50 days), the mice were anesthetized by intraperitoneal injection of 0.7% pento-barbital (10 μl/g), and average fluorescence intensity and distribution using a region of interest centered on the xenograft tumors were observed by a *in vivo* imaging system (IVIS Spectrum; Perkin Elmer). Then mice were sacrificed, and the weight and volume of the tumors were measured. The tumor volume was measured using the formula V = 3.14/6 × L × W × W, where W represents the width of the tumor and L represents the length of the tumor. The tumor tissues were fixed with 4% paraformaldehyde overnight, embedded in paraffin, and cut into 4 μm paraffin sections for subsequent experiments.

### Immunohistochemistry (IHC)

The sections cut from patient specimens or xenograft tumors were dewaxed, rehydrated, and then subjected to heat-induced epitope repair in 0.01 M sodium citrate buffer (pH 6.0). The endogenous peroxidase was blocked by 3% hydrogen peroxide bath for 15 min. After washing with Tris-buffered saline (TBS), the sections were incubated with primary antibody against POLE2 (bs-14356R; BIOSS; diluted 1:200), STC1 (cat. no. Ab229477; Abcam; diluted 1:200) or Ki67 (cat. no. Ab16667; Abcam; diluted 1:200) overnight at 4°C, followed by conjugation to the secondary antibody (cat. no. Ab6721; Abcam; diluted 1:400) and DAB staining, then sections were counterstained using hematoxylin, dehydrated, and sealed with neutral gum.

POLE2 staining was scored for the percentage of positive cells and the intensity of staining in the cytoplasm. The scoring system for intensity was: 0, no staining; 1, weak staining; 2, moderate staining; and 3, strong staining. The scoring system for the percentage of stained tumor cells was: 0, ≤0% stained cells; 1, 1–24% stained cells; 2, 25–49% stained cells; 3, 50–74% stained cells; and 4, ≥75% stained cells. A final score was the product of the staining intensity and the percentage of stained cells. A score of <4 was considered POLE2 low expression and a score ≥4 was considered POLE2 high expression.

### Hematoxylin-Eosin Staining Analysis

After dewaxed and rehydrated, the sections were stained with hematoxylin, rinsed with running water, stained with eosin, dehydrated by gradient ethanol, transparent with xylene, and sealed with neutral gum. Then the histopathology of the tumor was observed by light microscope.

### Microarray Procedure

Total RNA was isolated from ACHN cells stably transfected with shCtrl or shPOLE2 using TRIzol reagent. The quality and integrity of the extracted RNA was examined with Thermo NanoDrop 2000. Amplification, labeling, generation of cRNA, and hybridization were done by PERLAN Technologies (Warsaw, Poland) on Agilent’s human GE 4 × 44K v2 (G4845A) microarrays. The Limma R/Bioconductor package, version 3.22.7 (62) was used to perform microarray data background correction, quantile normalization, filtering of probes with low intensity in less than half of the samples, probe summarization at the gene level, quality control, principal component analysis (PCA), and statistical analysis of differentially expressed genes. Low-intensity probes were defined as those whose intensity was below a threshold set at 10% above the third quartile of negative probes. PCA was performed with the prcomp R function based on summarized expression data.

### Bioinformatics Analysis

#### Differentially Expressed Genes

Differential gene expression levels between shPOLE2-infected ACHN cells and shCtrl-infected ACHN cells were estimated with a linear model based on empirical Bayes distribution (Ritchie et al., 2015). *P*-values were corrected with the Benjamini-Hochberg algorithm (false discovery rate; FDR). The differentially expressed genes (DEGs) were identified based on | Fold Change| ≥ 1.3 and FDR < 0.05.

#### Pathway and Network Analysis by IPA

The list of differentially expressed genes between shPOLE2-infected ACHN cells and shCtrl-infected ACHN cells, containing gene identifiers and corresponding expression values, was uploaded into the IPA software (Qiagen). The “core analysis” function included in the software was used to interpret the differentially expressed data, which included biological processes, canonical pathways, upstream transcriptional regulators, and gene networks. Each gene identifier was mapped to its corresponding gene object in the Ingenuity Pathway Knowledge Base (IPKB).

### Human Apoptosis Antibody Array

The apoptosis signaling pathway was detected using the Human Apoptosis Antibody Array Kit (ab134001, Abcam). In brief, the cells were washed with PBS and lysed with lysis buffer at 2–8°C for 30 min. After spun down at 14000 × *g* for 10 min, the total extracted protein was diluted with the Array Diluent Buffer Kit (0.5 mg/mL). Each array of antibody membrane was blocked with blocking buffer for 30 min at room temperature, which incubated at 4°C overnight. Membranes were incubated with 1 × Biotin-conjugated Anti-Cytokines overnight at 4°C. Then, the membranes were incubated with 1.5 ml of Streptavidin-HRP at room temperature for 2 h. Protein was visualized using ChemiDoc XRS chemiluminescence detection (HyClone Pierce, Cat. No. 23225; Amersham, RPN2232) and imaging system. The density of the spots was quantitated using Quantity One software and normalized to the α–tubulin levels.

### Statistical Analysis

The data were presented as the mean ± SD. The correlation between POLE2 expression and the clinicopathological features of the patients was performed with the Chi-square test or Mann-Whitney *U* analysis, followed by Spearman’s rank correlation analysis. The overall survival (OS) curve was analyzed with the Kaplan-Meier method and was compared with a log-rank test. The quantitative data were compared using Mann-Whitney *U* test or Student’s *t*-test. *P*-values < 0.05 were considered statistically significant.

## Results

### POLE2 Level Is Upregulated and Associated With Various Clinicopathological Parameters in RCC

**FIGURE 1:**
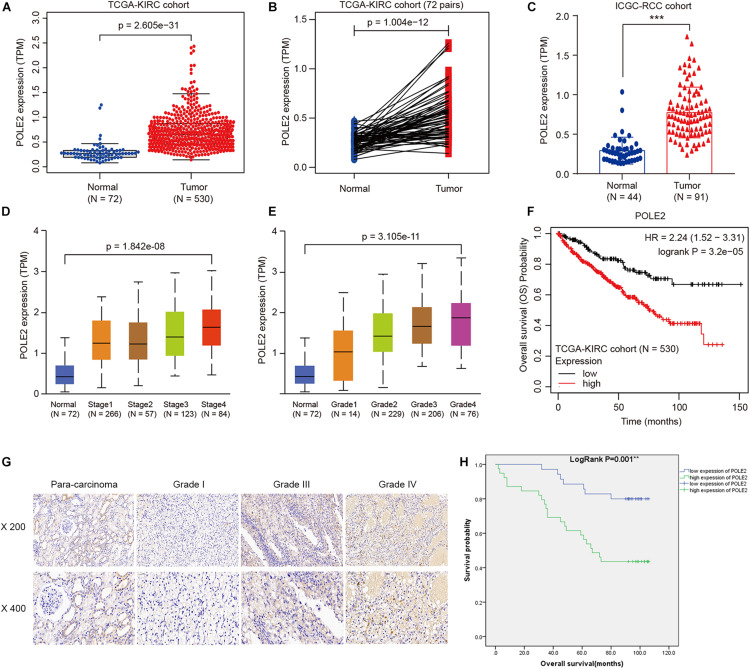
POLE2 level is upregulated and associated with various clinicopathological parameters in RCC. The POLE2 mRNA level in RCC was obtained from The Cancer Genome Atlas Kidney Clear Cell Carcinoma dataset (TCGA-KIRC; containing 72 normal tissues and 530 RCC tissues) and International Cancer Genome Consortium (ICGC; containing 44 normal tissues and 91 RCC tissues). The POLE2 mRNA levels were compared in different clinicopathological parameters: **(A,C)** Cancer vs. para-cancer, **(B)** Carcinoma vs. paired para-cancer, **(D)** TNM stage, and **(E)** G stage. **(F)** Kaplan-Meier curves of the overall survival of RCC patients with high or low POLE2 expression in TCGA-KIRC cohort. **(G)** Representative immunohistochemical staining for POLE2 in RCC and adjacent normal tissues. **(H)** Kaplan-Meier curves of the overall survival of RCC patients with high or low POLE2 expression. **P < 0.01, ***P < 0.001.

The mRNA expression levels of POLE2 in RCC cancer tissues and corresponding normal tissues of TCGA-KIRC and ICGC-RCC cohorts were investigated and the results pointed out that the expression of POLE2 in RCC tissues was significantly higher than that in normal tissues (Figures 1A–C). In addition, the RCC patients with higher level of POLE2 had higher pathological TNM stage and grade, as well as poor prognosis (Figures 1D–F). To verify the high POLE2 expression in RCC, the expression of POLE2 in clinical RCC specimens was detected using IHC. The results showed that the POLE2 expression in RCC tissues was significantly higher and increased with pathological grade progression compared with the normal tissues (Figure 1G, Supplementary Figure 1A, and Table 1). Among the RCC tissues, 45.8% cases were classified as POLE2-low, whereas 54.2% stained high for POLE2. According to Mann-Whitney *U* analysis, it was found that there were significant differences in pathological grade and pathological stage between the high and low POLE2 expression groups, yet there was no significant difference in age, gender and tumor size between two groups (Table 2). Then, Spearman’s rank correlation analysis showed that the expression of POLE2 was positively correlated with tumor grade, stage and pathological T indicating that the expression of POLE2 increased with the degree of tumor malignancy (Table 3). Besides, the Kaplan-Meier survival curve revealed that the OS and progression-free survival of patients with POLE2 high expression was significantly lower than that of patients with POLE2 low expression (Figure 1H and Supplementary Figure 1B), which suggested that the expression of POLE2 in RCC tissues was negatively associated with prognosis. Therefore, these results indicated that the POLE2 expression was downregulated in RCC and significantly associated with various clinicopathological parameters in RCC.

**TABLE 1:** POLE2 expression in renal cell carcinoma and para-carcinoma tissues detected by immunohistochemistry.

POLE2 expression	Tumor tissue		Para-carcinoma tissue		*P*-value
	Cases	Percentage	Cases	Percentage	
Low	38	45.8%	82	98.8%	0.000***
High	45	54.2%	1	1.2%	

***P < 0.001.

**TABLE 2:** Relationship between POLE2 expression and tumor characteristics in patients with renal cell carcinoma.

Features	No. of patients (*n* = 83)	POLE2 expression		*P*-value
		Low (*n* = 38)	High (*n* = 45)	
Age (years)				0.763
<59	40	19	21	
≥59	43	19	24	
Gender				0.679
Male	46	22	24	
Female	37	16	21	
Tumor size				0.146
≤5 cm	43	23	20	
>5 cm	40	15	25	
Grade				0.001**
I	31	20	11	
II	38	17	21	
III	13	1	12	
IV	1	0	1	
Stage				0.004**
1	61	34	27	
2	17	3	14	
3	3	0	3	
4	2	1	1	
Pathological T				0.011*
T1	62	34	28	
T2	17	3	13	
T3	4	1	3	

*P < 0.05, **P < 0.01.

**TABLE 3:** Relationship between POLE2 expression and tumor characteristics in patients with renal cell carcinoma.

POLE2		*P*-value
Grade	Pearson correlation	0.374
	Significance (double tailed)	0.000***
	N	83
Stage	Pearson correlation	0.334
	Significance (double tailed)	0.003**
	N	77
Pathological T	Pearson correlation	0.308
	Significance (double tailed)	0.006**
	N	79

**P < 0.01, ***P < 0.001.

### Knockdown of POLE2 Expression Inhibits RCC Cell Proliferation and Migration

**FIGURE 2:**
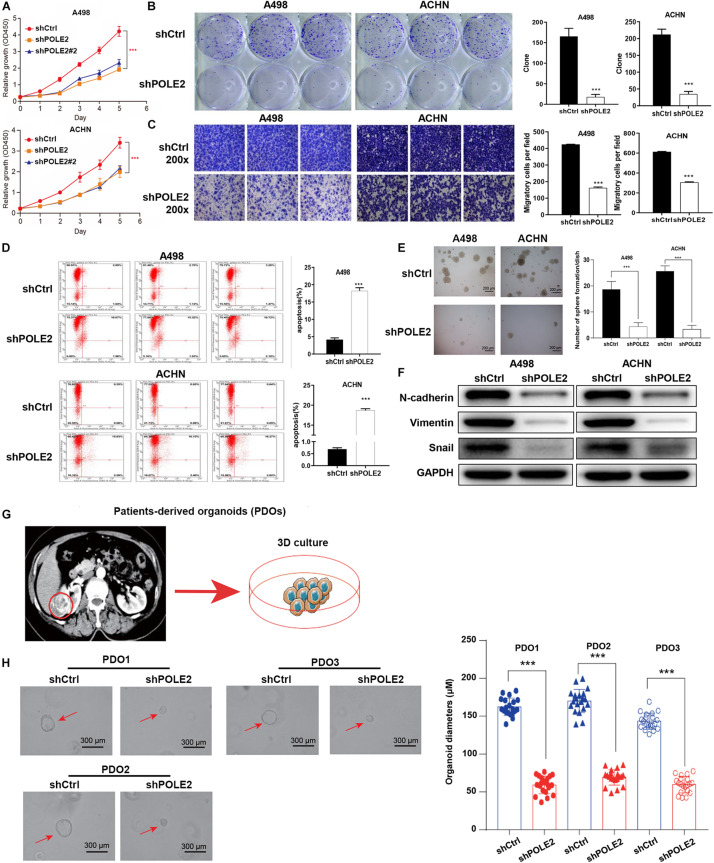
Knockdown of POLE2 expression inhibits RCC cell proliferation and migration. **(A)** The cell proliferation activity of A498 and ACHN cells infected with shCtrl or shPOLE2 lentivirus was detected by MTT assays. **(B)** The clone formation ability of A498 and ACHN cells infected with shCtrl or shPOLE2 lentivirus was detected by **(B)** clone formation assays. **(C)** The migration ability of A498 and ACHN cells infected with shCtrl or shPOLE2 lentivirus was detected by Transwell assays. **(D)** The apoptosis rate of A498 and ACHN cells infected with shCtrl or shPOLE2 lentivirus was measured using flow cytometry. **(E)** The clone formation ability of A498 and ACHN cells infected with shCtrl or shPOLE2 lentivirus was detected by soft agar colony formation assays. **(F)** The protein expression levels of N-cadherin, Vimentin and Snail in A498 and ACHN cells infected with shCtrl or shPOLE2 lentivirus was detected by Western blot, GAPDH served as loading control. **(G)** PDOs were generated from biopsies of RCC patients (red circle) and cultured. **(H)** Representative images of three different RCC organoids transfected with shCtrl or shPOLE2 lentivirus and quantification of organoid diameters. ***P < 0.001. The red arrow points to the organoid.

To investigate whether POLE2 played an important biological role in RCC, we successfully downregulated the expression of POLE2 in A498 and ACHN cells by lentivirus-induced RNAi (Supplementary Figure 2). The results showed that knockdown of POLE2 significantly inhibited proliferation, migration, and facilitated apoptosis of A498 and ACHN cells (Figures 2A–E and Supplementary Figure 3). Furthermore, the results of Western Blot found that knockdown of POLE2 downregulated the protein levels of N-cadherin, Vimentin and Snail both in A498 and ACHN cells, indicating the cell epithelial-mesenchymal transition was suppressed (Figure 2F). At same time, RCC organoid models was established to further verify the potential clinical value of POLE2. The results revealed that knockdown of POLE2 significantly inhibited cell proliferation in the RCC organoids generated from three different RCC patients (Figures 2G,H). Thus, these data suggested that knockdown of POLE2 could inhibit RCC cell proliferation and migration.

### Knockdown of POLE2 Attenuates Tumorigenesis and Tumor Growth of ACHN Cells *in vivo*

**FIGURE 3:**
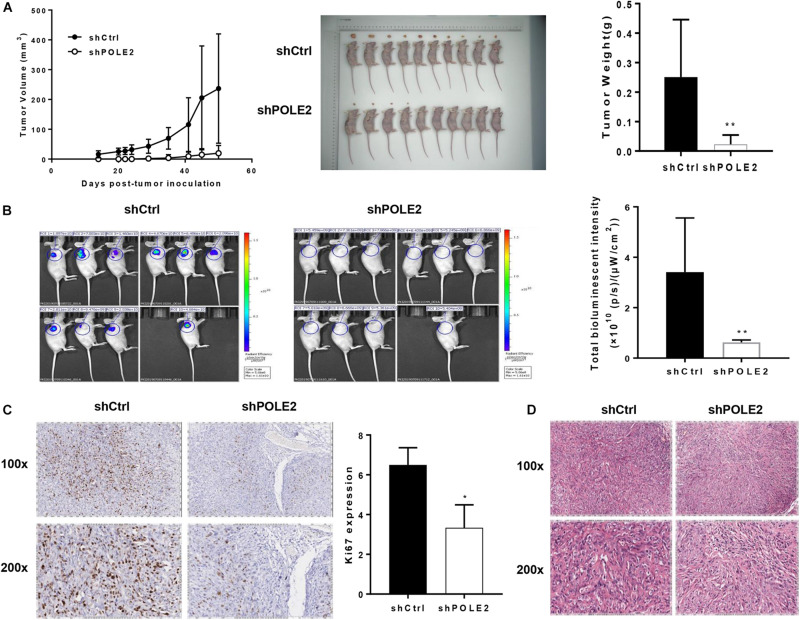
Knockdown of POLE2 attenuated tumorigenesis and tumor growth of ACHN cells *in vivo*. **(A)** The growth curves of xenografts from ACHN cells infected with shCtrl or shPOLE2 lentivirus, and the tumor weight examined at the ending point. **(B)** Fluorescent intensity of the xenograft model at the ending point. **(C)** Immunohistochemical staining for proliferation marker protein Ki-67 protein in the xenograft tumors. **(D)** HE staining in the xenograft tumors. *P < 0.05, **P < 0.01.

Then, the pro-oncogenesis of POLE2 *in vivo* was analyzed by seeding the ACHN cells infected with shCtrl or shPOLE2 lentivirus into 4 weeks female nude mice. The results showed that the xenograft volume of two groups grew in a time-dependent manner, but the xenografts in shCtrl group grew significantly faster than that in shPOLE2 group. At the ending point, all of xenografts were collected. The xenografts in shCtrl group were obviously bigger and heavier than that in shPOLE2 group (Figure 3A). The results of *in vivo* imaging verified that the fluorescent intensity was diminished in the xenograft of the shPOLE2 group compared with that in the shCtrl group (Figure 3B), indicating that POLE2 played a pivotal role in tumorigenesis and tumor growth of ACHN cells. Besides, as shown in Figure 3C, the Ki67 protein expression in shPOLE2 group was reduced compared with the shCtrl group, and the results of HE staining pointed out that there were obvious differences in the pathological morphology of the tumor in the two groups (Figure 3D). The above experiments proved that knockdown of POLE2 can inhibit the tumorigenesis of ACHN cells in mice, suggesting the promotion of POLE2 in RCC.

### POLE2 Interacts With STC1

**FIGURE 4:**
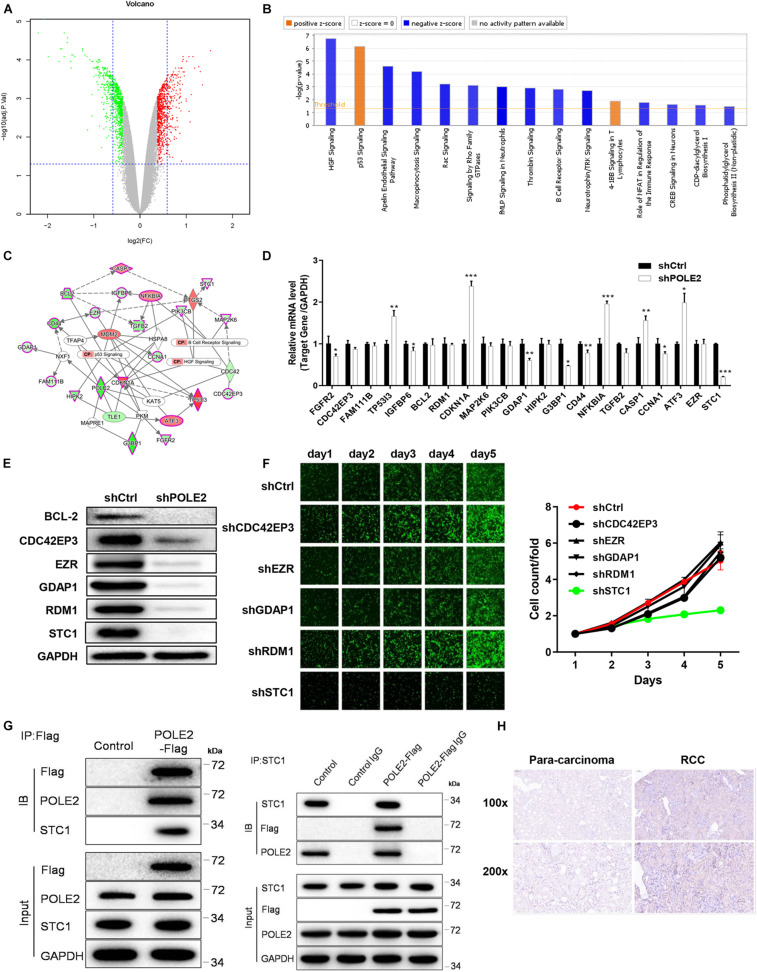
POLE2 interacts with STC1. **(A)** The volcano map of DEGs between normal ACHN cells and knock-down POLE2 ACHN cells. **(B)** Classic pathway enrichment analysis. **(C)** Ingenuity Pathway Analysis (IPA)-identified interaction network between POLE2 and the pathways p53 Signaling, B Cell Receptor Signaling, and HGF Signaling. **(D,E)** The expression levels of downstream genes related to POLE2 were detected by **(D)** qRT-PCR and **(E)** Western blot. **(F)** The cell proliferation activity of ACHN cells infected with shCtrl, sh CDC42EP3, shEZR, shGDAP1, shRDM1 or shSTC1 lentivirus was detected by HCS Cell Proliferation Assay. **(G)** The interaction of POLE2 and STC1 in ACHN cells were examined by Co-IP experiment. **(H)** Representative immunohistochemical staining for STC1 in RCC and adjacent normal tissues. *P < 0.05, **P < 0.01, ***P < 0.001.

In order to clarify the molecular mechanism of POLE2 in tumorigenesis and development of RCC, the DEGs in normal ACHN cells and POLE2 knockdown ACHN cells were screened by expression profile sequencing analysis, and their expression was verified by qRT-PCR and western blot. As depicted in Figures 4A, 517 upregulated and 505 downregulated DEGs were identified from the comparative analysis on shCtrl and shPOLE2 groups. To characterize these DEGs, we next performed the enrichment analysis by using IPA. The results showed that these DEGs were highly significant overlap of 221 canonical pathways, in which HGF Signaling, Apelin Endothelial Signaling Pathway, Macropinocytosis Signaling, Rac Signaling, Signaling by Rho Family GTPases, fMLP Signaling in Neutrophils, Thrombin Signaling, B Cell Receptor Signaling, Neurotrophin/TRK Signaling, Role of NFAT in Regulation of the Immune Response, CREB Signaling in Neurons, CDP-diacylglycerol Biosynthesis I, and Phosphatidylglycerol Biosynthesis II (Non-plastidic) pathways were significantly inhibited (Z-score ≤ −2), while p53 Signaling and 4-1BB Signaling in T Lymphocytes pathways were significantly activated (Z-score ≥ 2) (Figure 4B). Then the interaction network between significantly enriched pathways (p53 Signaling, B Cell Receptor Signaling, and HGF Signaling pathway genes) and the target gene POLE2 were constructed by IPA. As shown in Figure 4C, POLE2 indirectly affected downstream genes that might be associated with p53 Signaling, B Cell Receptor Signaling, and HGF Signaling pathways through genes such as NXF1, PKM, and KAT5, including ATF3, BCL2, CASP1, CCNA1, CD44, CDC42EP3, CDKN1A, EZR, FAM111B, FGFR2, G3BP1, GDAP1, HIPK2, IGFBP6, MAP2K6, NFKBIA, PIK3CB, STC1, TGFB2, TP53I3, etc. These downstream genes related to POLE2 were screened and the expression levels were detected by qRT-PCR and western blot. The results of qRT-PCR revealed that compared with shCtrl group, the mRNA expression levels of FGFR2, IGFBP6, GDAP1, G3BP1, CD44, CCNA1 and STC1 were significantly reduced, while the mRNA expression levels of TP53I3, CDKN1A, NFKBIA, CASP1, and ATF3 were obviously increased (Figure 4D). In addition, the results of western blot also found that the protein expression levels of BCL2, CDC42EP3, EZR, GDAP1, RDM1, and STC1 were significant decreased (Figure 4E). Then, we knockdown the expression of these proteins and found that the RCC cell proliferation was significantly inhibited after STC1 knockdown (Figure 4F), Thus, STC1 was selected as a candidate downstream gene, and the interaction of POLE2 and STC1 was detected using Co-IP method. As shown in Figure 4G, POLE2 protein interacted with STC1 protein in ACHN cells. Then, the differential expression of STC1 in RCC tissue was verified from clinical tissue samples. The results revealed that STC1 was highly expressed in RCC tissues compared with the adjacent normal tissues (Figure 4H), clarifying that POLE2-STC1 might play a role in promoting the occurrence and development of RCC.

### POLE2 Promotes RCC Cell Proliferation and Migration by Regulating STC1

**FIGURE 5:**
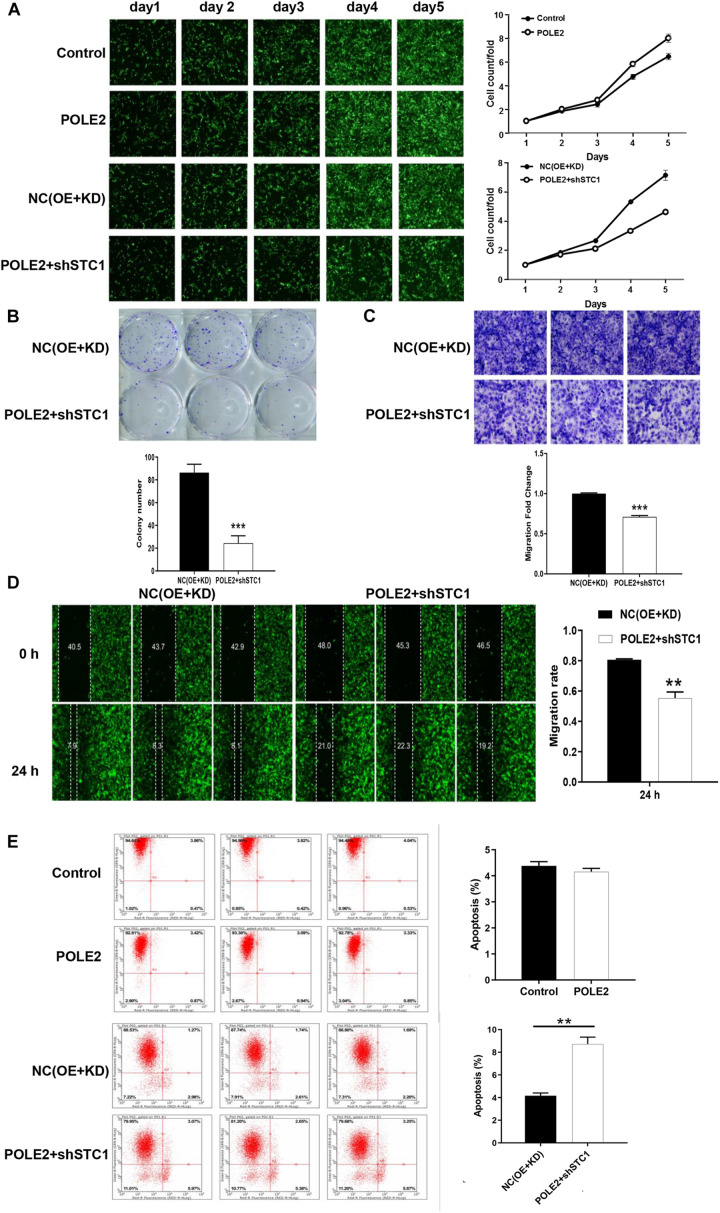
POLE2 promotes RCC cell proliferation and migration by regulating STC1. **(A)** The cell proliferation activity of control, POLE2, NC(OE + KD), POLE2 + shSTC1 groups was detected by HCS Cell Proliferation Assay. **(B)** The clone formation ability of NC(OE + KD) and POLE2 + shSTC1 groups was detected by clone formation assays. **(C,D)** The migration ability of NC(OE + KD) and POLE2 + shSTC1 groups was evaluated by **(C)** Transwell assays and **(D)** wound-healing assay. **(E)** The apoptosis rate of control, POLE2, NC(OE + KD), POLE2 + shSTC1 group was measured using flow cytometry. **P < 0.01, ***P < 0.001.

Furthermore, the upstream and downstream relationship of POLE2-STC1 and their effect on RCC was determined by downstream gene function rescue test. We constructed overexpressing POLE2 ACHN cells and knockdown STC1 with overexpressing POLE2 ACHN cells, then detected changes in their biological functions. The results showed that overexpression of POLE2 promoted the proliferation of ACHN cells and inhibited the apoptosis (Figures 5A,E), while knockdown of STC1 in overexpressing POLE2 ACHN cells inhibited the proliferation and migration of ACHN cells, and promoted their apoptosis (Figures 5A–E).

### The Underlying Mechanism of POLE2 on Apoptosis of A498 Cells

**FIGURE 6:**
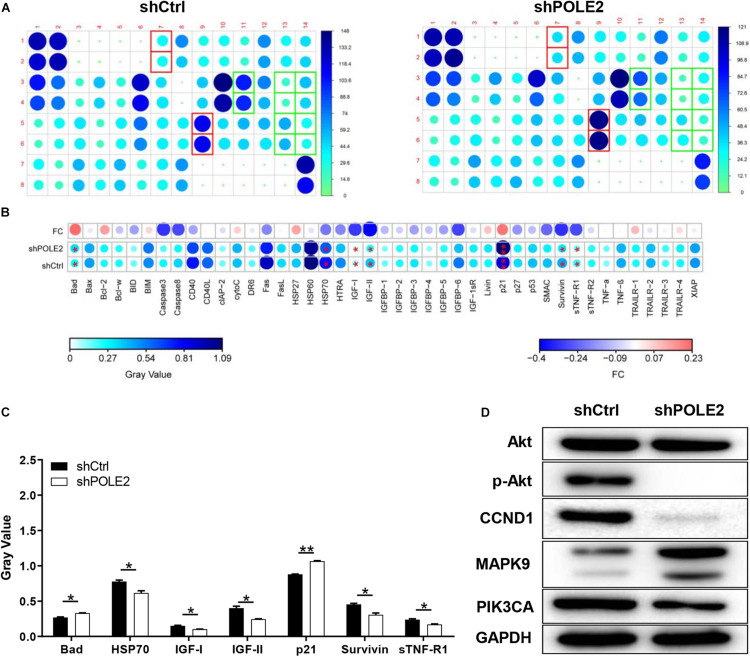
The underlying mechanism of POLE2 on apoptosis of A498 cells. **(A)** The apoptosis relative proteins were detected by human apoptosis antibody array in A498 cells with or without POLE2 knockdown. **(B)** Differences in human apoptotic antibody array were analyzed in A498 cells with or without POLE2 knockdown. **(C)** The gray values of differentially expressed proteins in A498 cells with or without POLE2 knockdown. **(D)** Cancer-related pathway proteins were confirmed by western blot in A498 cells with or without POLE2 knockdown, GAPDH served as loading control. *P < 0.05, **P < 0.01.

Then, the human apoptosis antibody array and western blot was applied to explore the potential mechanism of POLE2 on RCC apoptosis. The results pointed out that knockdown of POLE2 significantly upregulated the expression levels of Bad and p21 while the expression levels of HSP70, IGF-I, IGF-II, survivin, and sTNF-R1 were significantly down (Figures 6A–C). Western blot analysis also showed that knockdown of POLE2 inhibited the expression levels of cancer-related pathway proteins including p-Akt, CCND1, and PIK3CA, while the expression of MAPK9 was promoted (Figure 6D).

## Discussion

POLE2 is a gene involved in DNA replication process, which can repair errors in the DNA replication process and reduce the occurrence of gene mutations (Burgers, 1998). Previous studies showed that POLE2 is overexpressed in many cancers, such as lung adenocarcinoma, breast cancer, colorectal cancer and esophageal squamous cell carcinoma (ESCC), and it can promote tumor development (Spier et al., 2015; Li et al., 2018; Pearlman et al., 2019; Zhu et al., 2020). In the present study, POLE2 was also high expressed in RCC and associated with tumor poor prognosis of RCC patients, which was confirmed in TCGA, ICGC, and clinical RCC specimens. Besides, we found that knockdown of POLE2 inhibited RCC cell proliferation, migration and promoted apoptosis *in vitro*, as well as had a negative effect on tumor occurrence and development *in vivo*, which was consistent with other research results (Su et al., 2020). Therefore, POLE2 may by served as a therapeutic target and potential prognostic factor for the treatment of RCC.

**FIGURE 7:**
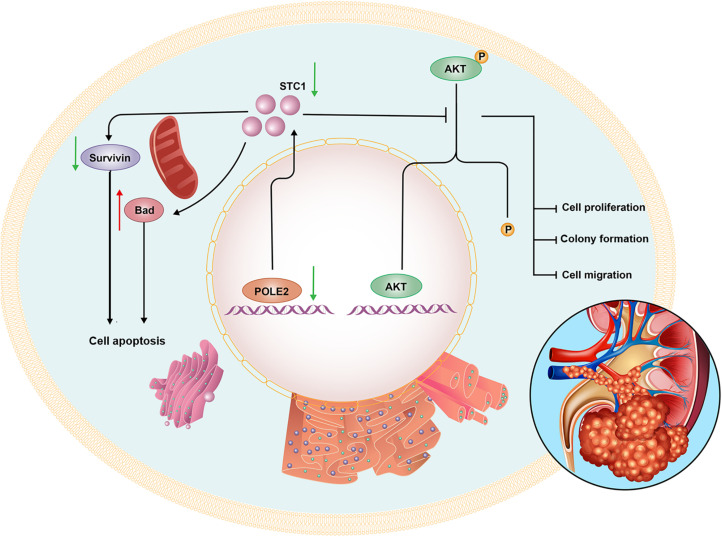
The mechanism diagram of POLE2 involved in the biological function of RCC.

Then, we further investigated the underlying mechanisms of functions of POLE2 in RCC. By sequencing and analysis of gene expression profiles, the gene STC1 was screened and its protein expression was verified in the RCC specimens. STC1 is a known oncogene that plays a role in many cancers. It was pointed out in the literature that STC1 affected the occurrence and development of ovarian cancer (Zhang et al., 2019), and could activate phosphorylation of Akt thereby affecting epithelial-mesenchymal transition (EMT) (Yang et al., 2019). Besides, STC1 could affect the metastasis of glioma through the TGF-β/SMAD4 pathway (Xiong and Wang, 2019), and affect the metastasis of liver cancer through the JNK pathway (Chan et al., 2017). Some studied found that exogenous STC-1 could promote the RCC proliferation by reducing the levels of HIF-1α and Ca^2+^ (Zhu et al., 2014; Yang Q. et al., 2015). Additionally, Ma et al. (2015) pointed out that the expression of STC1 in clear cell renal cell carcinoma (ccRCC) was significantly upregulated, especially in metastatic ccRCC, meanwhile knockdown of STC1 expression inhibited cell proliferation, migration and invasion, as well as damage EMT of ccRCC, which was consistent with our results. The co-IP experiment in this study determined that there was protein interaction between POLE-STC1. Besides, it was verified through rescue experiment that STC1 was downstream of POLE2, and it also had an impact on cell function, which was similar to POLE2. Therefore, we concluded that POLE2 regulated STC1 to promote the occurrence and development of RCC (Figure 7).

Furthermore, the human apoptosis antibody array was used to explore the mechanism of POLE2 regulating cell apoptosis. The results demonstrated that the expression levels of related genes in the human apoptosis signaling pathway such as Bad and p21 were significantly upregulated, while the expression levels of HSP70, IGF-I, IGF-II, Survivin, and sTNF-R1 were downregulated. Bad is a member of Bcl-2 family and is an important regulatory component of the intrinsic cell death machinery (Schürmann et al., 2000). p21 combined with p53 can regulate the cell apoptosis and invasion by targeting Bcl-2 proteins (Kim et al., 2017). HSP70 is usually overexpressed in RCC, which is involved in apoptotic cell death and regulation of cell proliferation (Atkins et al., 2005). IGF-I, IGF-II, Survivin, and sTNF-R1 are all antiapoptotic factors and participate in the occurrence and progression of RCC (Rosendahl et al., 2008; Sánchez-Lázaro et al., 2012; Carew et al., 2015; Solarek et al., 2019). Thus, we suggested that POLE2 involved in RCC cell apoptosis by regulating various apoptosis-related factors. Zhu et al. (2020) also confirmed that POLE2 was participated in ESCC apoptosis induction via upregulating pro-apoptotic proteins (Bax, Caspase3, CD40L, FasL, IGFBP-5, and P21) and downregulating anti-apoptotic proteins (CLAP-2, IGF-I, and sTNF-R2). Besides, they found that the expression of Akt, p-Akt, Cyclin D1, and PIK3CA were decreased, while the expression of MAPK9 was increased after POLE2 knockdown in ESCC, which was similar to our results. However, different from the study of Zhu et al. (2020), the downregulation of POLE2 in this study only inhibited the phosphorylation of Akt, but had no significant effect on the expression of Akt. The PI3K/Akt signaling pathway is dysregulated in a wide range of tumors. This pathway regulates cell proliferation and survival, and is closely related to tumor invasion and metastasis. Activated Akt can activate or inhibit downstream target proteins Bad and p21, thereby regulating cell proliferation, apoptosis and migration. Guo et al. (2015) claimed that the PI3K/AKT pathway was modestly mutated but highly activated in RCC, which may be a promising drug target for RCC treatment. CCND1, a G1 phase related protein, was found to be upregulated in RCC and was a good biomarker and therapeutic target for RCC tumor progression (Karim et al., 2016). PIK3CA is a common mutation in ccRCC and predicts a poor prognosis in RCC (Bodnar et al., 2015; D’Avella et al., 2020). MAPK9 can effectively promote cancer cell apoptosis and hinder cancer cell invasion and metastasis. The study found that RCC patients with positive MAPK9 expression have a better local prognosis and longer overall survival time after sorafenib treatment (Yang P. et al., 2015). Taken these together, it was suggested that knockdown of POLE2 might attenuate proliferation and migration via inducing apoptosis by regulating various apoptosis-associated factors and PI3K/AKT, CCND1 signal pathway (Figure 7).

## Conclusion

Knockdown of POLE2 inhibits RCC cell proliferation and migration via regulating STC1, which may serve as a potential target for RCC therapy.

## Data Availability Statement

The raw data supporting the conclusions of this article will be made available by the authors, without undue reservation.

## Ethics Statement

The studies involving human participants were reviewed and approved by the Ethics Committee of Shanghai Ruijin Hospital. The patients/participants provided their written informed consent to participate in this study. The animal study was reviewed and approved by the Ethics Committee of Shanghai Ruijin Hospital.

## Author Contributions

CZ and HH conceived and designed the study. YS, LG, XW, and DH contributed to the experiment and analysis of the data. CZ, YS, and XX wrote the first draft of manuscript. DX and HH critically revised the manuscript. All authors read and approved the final manuscript.

## Conflict of Interest

The authors declare that the research was conducted in the absence of any commercial or financial relationships that could be construed as a potential conflict of interest.

## Footnotes

1 https://portal.gdc.cancer.gov/

2 https://icgc.org/

## References

[B1] Atkins D. Lichtenfels R. Seliger B. (2005). Heat shock proteins in renal cell carcinomas. *Contrib. Nephrol.* 148 35–56. 10.1159/000086042 15912026

[B2] Bodnar L. Stec R. Cierniak S. Synowiec A. Wcisło G. Jesiotr M. (2015). Clinical usefulness of PI3K/Akt/mTOR genotyping in companion with other clinical variables in metastatic renal cell carcinoma patients treated with everolimus in the second and subsequent lines. *Ann. Oncol.* 26 1385–1389. 10.1093/annonc/mdv166 25962440

[B3] Bray F. Ferlay J. Soerjomataram I. Siegel R. L. Torre L. A. Jemal A. (2018). Global cancer statistics 2018: GLOBOCAN estimates of incidence and mortality worldwide for 36 cancers in 185 countries. *CA Cancer J. Clin.* 68 394–424. 10.3322/caac.21492 30207593

[B4] Burgers P. M. (1998). Eukaryotic DNA polymerases in DNA replication and DNA repair. *Chromosoma* 107 218–227. 10.1007/s004120050300 9745046

[B5] Carew J. S. Espitia C. M. Zhao W. Mita M. M. Mita A. C. Nawrocki S. T. (2015). Targeting Survivin Inhibits Renal Cell Carcinoma Progression and Enhances the Activity of Temsirolimus. *Mol. Cancer Ther.* 14 1404–1413. 10.1158/1535-7163.mct-14-1036 25808836

[B6] Chan K. K. Leung C. O. Wong C. C. Ho D. W. Chok K. S. Lai C. L. (2017). Secretory Stanniocalcin 1 promotes metastasis of hepatocellular carcinoma through activation of JNK signaling pathway. *Cancer Lett.* 403 330–338. 10.1016/j.canlet.2017.06.034 28688970

[B7] Chen W. Zheng R. Baade P. D. Zhang S. Zeng H. Bray F. (2016). Cancer statistics in China, 2015. *CA Cancer J. Clin.* 66 115–132. 10.3322/caac.21338 26808342

[B8] Chubb D. Broderick P. Dobbins S. E. Frampton M. Kinnersley B. Penegar S. (2016). Rare disruptive mutations and their contribution to the heritable risk of colorectal cancer. *Nat. Commun.* 7:11883. 10.1038/ncomms11883 27329137 PMC4917884

[B9] D’Avella C. Abbosh P. Pal S. K. Geynisman D. M. (2020). Mutations in renal cell carcinoma. *Urol Oncol.* 38 763–773. 10.1016/j.urolonc.2018.10.027 30478013

[B10] Guo H. German P. Bai S. Barnes S. Guo W. Qi X. (2015). The PI3K/AKT Pathway and Renal Cell Carcinoma. *J. Genet. Genomics* 42 343–353. 10.1016/j.jgg.2015.03.003 26233890 PMC4624215

[B11] Hartmann E. Fernàndez V. Moreno V. Valls J. Hernández L. Bosch F. (2008). Five-gene model to predict survival in mantle-cell lymphoma using frozen or formalin-fixed, paraffin-embedded tissue. *J. Clin. Oncol.* 26 4966–4972. 10.1200/jco.2007.12.0410 18606985

[B12] Jiang Z. Chu P. G. Woda B. A. Liu Q. Balaji K. C. Rock K. L. (2008). Combination of quantitative IMP3 and tumor stage: a new system to predict metastasis for patients with localized renal cell carcinomas. *Clin. Cancer Res.* 14 5579–5584. 10.1158/1078-0432.ccr-08-0504 18765551

[B13] Karim S. Al-Maghrabi J. A. Farsi H. M. Al-Sayyad A. J. Schulten H. J. Buhmeida A. (2016). Cyclin D1 as a therapeutic target of renal cell carcinoma- a combined transcriptomics, tissue microarray and molecular docking study from the Kingdom of Saudi Arabia. *BMC Cancer* 16(Suppl. 2):741. 10.1186/s12885-016-2775-2 27766950 PMC5073805

[B14] Kim E. M. Jung C. H. Kim J. Hwang S. G. Park J. K. Um H. D. (2017). The p53/p21 Complex Regulates Cancer Cell Invasion and Apoptosis by Targeting Bcl-2 Family Proteins. *Cancer Res.* 77 3092–3100. 10.1158/0008-5472.can-16-2098 28377455

[B15] Li J. Wang J. Yu J. Zhao Y. Dong Y. Fan Y. (2018). Knockdown of POLE2 expression suppresses lung adenocarcinoma cell malignant phenotypes in vitro. *Oncol. Rep.* 40 2477–2486. 10.3892/or.2018.6659 30132567 PMC6151888

[B16] Ma X. Gu L. Li H. Gao Y. Li X. Shen D. (2015). Hypoxia-induced overexpression of stanniocalcin-1 is associated with the metastasis of early stage clear cell renal cell carcinoma. *J. Transl. Med.* 13:56. 10.1186/s12967-015-0421-4 25740019 PMC4337255

[B17] Pearlman A. Rahman M. T. Upadhyay K. Loke J. Ostrer H. (2019). Ectopic Otoconin 90 expression in triple negative breast cancer cell lines is associated with metastasis functions. *PLoS One* 14:e0211737. 10.1371/journal.pone.0211737 30763339 PMC6375562

[B18] Ritchie M. E. Phipson B. Wu D. Hu Y. Law C. W. Shi W. (2015). limma powers differential expression analyses for RNA-sequencing and microarray studies. *Nucleic Acids Res.* 43:e47. 10.1093/nar/gkv007 25605792 PMC4402510

[B19] Rosendahl A. H. Holly J. M. Celander M. Forsberg G. (2008). Systemic IGF-I administration stimulates the in vivo growth of early, but not advanced, renal cell carcinoma. *Int. J. Cancer* 123 1286–1291. 10.1002/ijc.23642 18561321

[B20] Sánchez-Lázaro I. J. Almenar-Bonet L. Romero-Pelechano A. Portoles-Sanz M. Martínez-Dolz L. Roselló-Lleti E. (2012). Serum markers of apoptosis in the early period of heart transplantation. *Biomarkers* 17 254–260. 10.3109/1354750x.2012.664168 22435528

[B21] Schürmann A. Mooney A. F. Sanders L. C. Sells M. A. Wang H. G. Reed J. C. (2000). p21-activated kinase 1 phosphorylates the death agonist bad and protects cells from apoptosis. *Mol. Cell Biol.* 20 453–461. 10.1128/mcb.20.2.453-461.2000 10611223 PMC85099

[B22] Solarek W. Koper M. Lewicki S. Szczylik C. Czarnecka A. M. (2019). Insulin and insulin-like growth factors act as renal cell cancer intratumoral regulators. *J. Cell Commun. Signal.* 13 381–394. 10.1007/s12079-019-00512-y 30929166 PMC6732138

[B23] Spier I. Holzapfel S. Altmüller J. Zhao B. Horpaopan S. Vogt S. (2015). Frequency and phenotypic spectrum of germline mutations in POLE and seven other polymerase genes in 266 patients with colorectal adenomas and carcinomas. *Int. J. Cancer* 137 320–331. 10.1002/ijc.29396 25529843

[B24] Su Y. Li C. Liu K. Wei L. Li D. Wang W. (2020). *Upregulation of Pole2 Promotes Clear Cell Renal Cell Carcinoma Progression via AKT/mTOR Pathway and Predicts a Poor Prognosis.* Preprint.

[B25] Vlachogiannis G. Hedayat S. Vatsiou A. Jamin Y. Fernández-Mateos J. Khan K. (2018). Patient-derived organoids model treatment response of metastatic gastrointestinal cancers. *Science* 359 920–926. 10.1126/science.aao2774 29472484 PMC6112415

[B26] Wu Z. Wang Y. M. Dai Y. Chen L. A. (2020). POLE2 Serves as a Prognostic Biomarker and Is Associated with Immune Infiltration in Squamous Cell Lung Cancer. *Med. Sci. Monit.* 26:e921430. 10.12659/msm.921430 32304567 PMC7191965

[B27] Xiong Y. Wang Q. (2019). STC1 regulates glioblastoma migration and invasion via the TGF-β/SMAD4 signaling pathway. *Mol. Med. Rep.* 20 3055–3064. 10.3892/mmr.2019.10579 31432189 PMC6755173

[B28] Yang P. Xue Q. Yan F. Shi F. Zheng W. Wang F. (2015). Relationship between MAPK9 expression in renal clear cell carcinoma and the efficacy of sorafenib targeted therapy. *Prog. Modern Biomed.* 15, 4298–4302.

[B29] Yang Q. Gu J. Shi J. Jia B. Gu C. Zhang Y. (2015). Influence of STC-1 on growth regulation of renal carcinoma cells. *J. Guiyang Med. Coll.* 40, 1043–1046, 1050.

[B30] Yang Y. Yin S. Li S. Chen Y. Yang L. (2019). Stanniocalcin 1 in tumor microenvironment promotes metastasis of ovarian cancer. *Onco. Targets Ther.* 12 2789–2798. 10.2147/ott.s196150 31114228 PMC6489642

[B31] Zekri A. R. Hassan Z. K. Bahnassy A. A. Khaled H. M. El-Rouby M. N. Haggag R. M. (2015). Differentially expressed genes in metastatic advanced Egyptian bladder cancer. *Asian Pac. J. Cancer Prev.* 16 3543–3549. 10.7314/apjcp.2015.16.8.3543 25921176

[B32] Zhang C. Wang B. Wang X. Sheng X. Cui Y. (2019). Sevoflurane inhibits the progression of ovarian cancer through down-regulating stanniocalcin 1 (STC1). *Cancer Cell Int.* 19:339. 10.1186/s12935-019-1062-0 31889892 PMC6916020

[B33] Zhao J. J. Chen P. J. Duan R. Q. Li K. J. Wang Y. Z. Li Y. (2014). Up-regulation of miR-630 in clear cell renal cell carcinoma is associated with lower overall survival. *Int. J. Clin. Exp. Pathol.* 7 3318–3323. 25031755 PMC4097229

[B34] Zhou Q. Effati R. Talvinen K. Pospiech H. Syväoja J. E. Collan Y. (2008). Genomic changes of the 55 kDa subunit of DNA polymerase epsilon in human breast cancer. *Cancer Genomics Proteomics* 5 287–292. 19129559

[B35] Zhu Y. Chen G. Song Y. Chen Z. Chen X. (2020). POLE2 knockdown reduce tumorigenesis in esophageal squamous cells. *Cancer Cell Int.* 20:388. 10.1186/s12935-020-01477-4 32831648 PMC7422519

[B36] Zhu Z. H. Gu J. Zhang Y. C. Yang Q. T. Yang Y. A. Wang N. (2014). [STC-1 is involved in anti-hypoxia proliferative balance of renal cancer cells by down-regulation of intracellular Ca2+ and HIF-1α levels]. *Zhejiang Da Xue Xue Bao Yi Xue Ban* 43 528–534. 25372636 10.3785/j.issn.1008-9292.2014.09.007

