# Supplementary figures and images for: Targeting POLE2 Creates a Novel Vulnerability in Renal Cell Carcinoma via Modulating Stanniocalcin 1
Source: Front Cell Dev Biol. 2021 Feb 11;9:622344. doi: 10.3389/fcell.2021.622344 (PMC7905105; doi:10.3389/fcell.2021.622344)

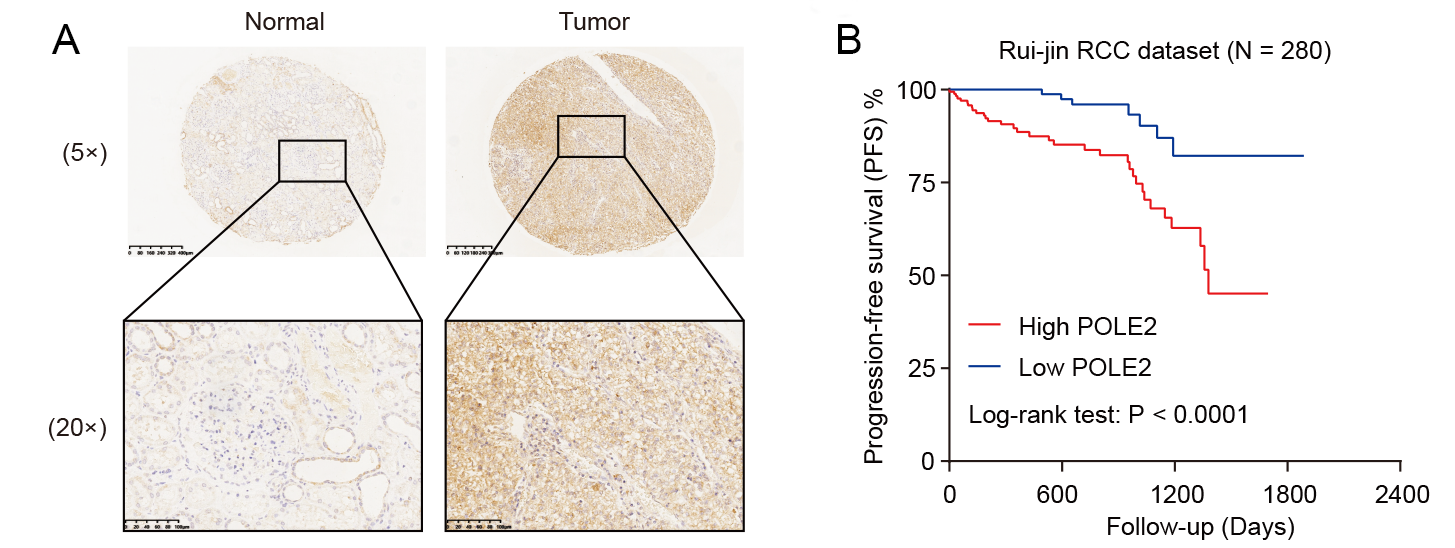

Supplement: Supplementary file 1 [file Image_1.TIF]

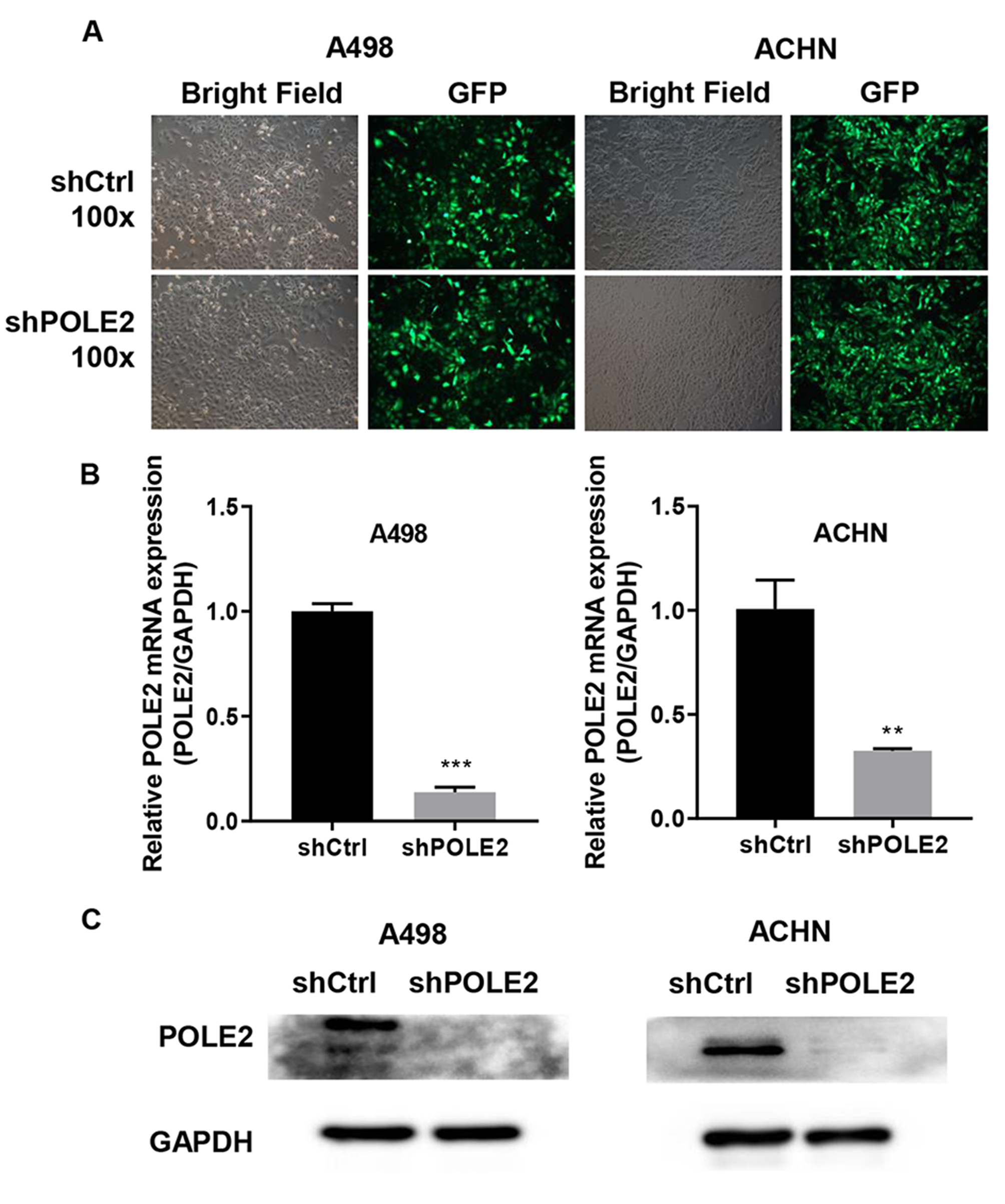

Supplement: Supplementary file 2 [file Image_2.TIF]

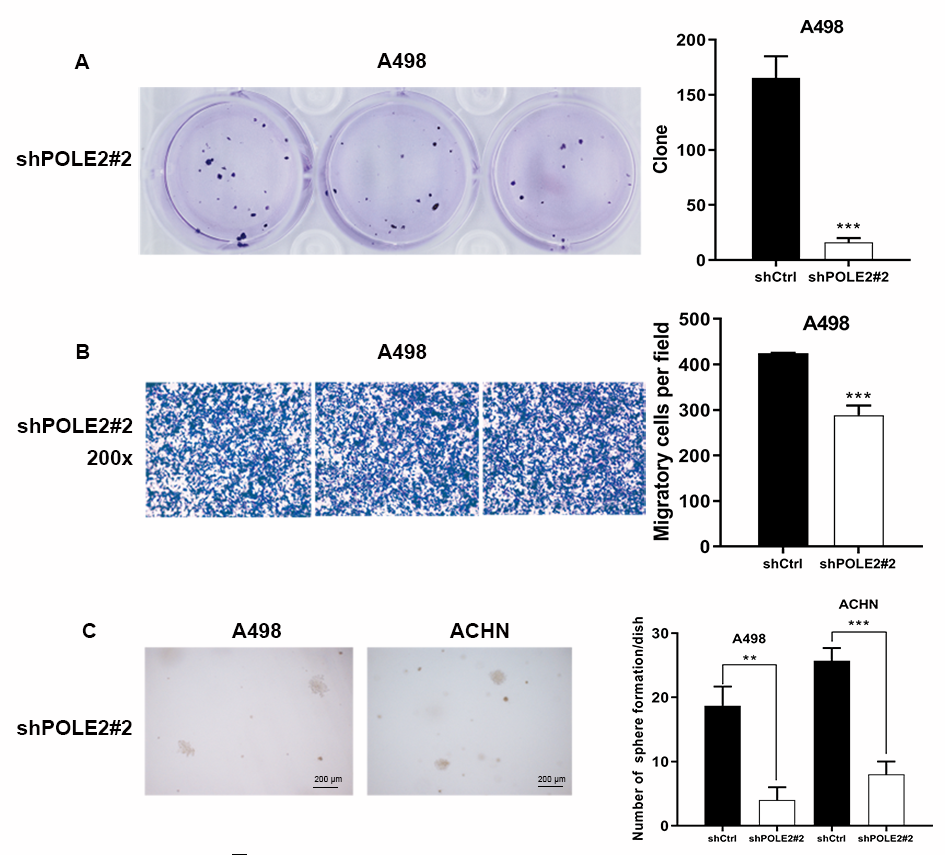

Supplement: Supplementary file 3 [file Image_3.TIF]
