# Supplementary material for: Targeting POLE2 Creates a Novel Vulnerability in Renal Cell Carcinoma via Modulating Stanniocalcin 1
Source: Front Cell Dev Biol. 2021 Feb 11;9:622344. doi: 10.3389/fcell.2021.622344 (PMC7905105; doi:10.3389/fcell.2021.622344)
Supplement: Supplementary file 4 [file Table_1.DOCX]

Supplementary Material

# Supplementary Tables

Table S1: PCR primers and length of amplification

| Primer | Primer sequences | | Product length（bp） |
| --- | --- | --- | --- |
| POLE2 | Primer F | 5'-TGAGAAGCAACCCTTGTCATC-3' | 84 |
|  | Primer R | 5'-TCATCAACAGACTGACTGCATTC-3' |  |
| GAPDH | Primer F | 5'-CGGATTTGGTCGTATTGGG-3' | 229 |
|  | Primer R | 5'-GATTTTGGAGGGATCTCGC-3' |  |
| FGFR2 | Primer F | 5'-GTATGAACTTCCAGAGGACCCA-3' | 98 |
|  | Primer R | 5'-CCATGACCACTTGCCCAAA-3' |  |
| CDC42EP3 | Primer F | 5'-AGCAGTCTGTTGGAGAATGGG-3' | 227 |
|  | Primer R | 5'-AGGAGGGAACCTGTAAGGTCAG-3' |  |
| FAM111B | Primer F | 5'-CCAGACAATTCCCAGGATTAGA-3' | 110 |
|  | Primer R | 5'-TAGCATACCGCCTACCCAGA-3' |  |
| TP53I3 | Primer F | 5'-TTTGCTGAGGTCTAGGGACAAT-3' | 141 |
|  | Primer R | 5'-TGGATTTCGGTCACTGGGTAG-3' |  |
| IGFBP6 | Primer F | 5'-GAATCCAGGCACCTCTACCA-3' | 94 |
|  | Primer R | 5'-CACTGAGTCCAGATGTCTACGG-3' |  |
| BCL2 | Primer F | 5'-TGTGGCCTTCTTTGAGTTCG-3' | 148 |
|  | Primer R | 5'-ATCCCAGCCTCCGTTATCCT-3' |  |
| RDM1 | Primer F | 5'-GCCCATCCTGGTTTCTATGC-3' | 150 |
|  | Primer R | 5'-GGCTTGATGTTGAACTGCCTTA-3' |  |
| CDKN1A | Primer F | 5'-TCTTGTACCCTTGTGCCTCG-3' | 105 |
|  | Primer R | 5'-GAAATCTGTCATGCTGGTCTGC-3' |  |
| MAP2K6 | Primer F | 5'-TCCGAGCCACAGTAAATAGCC-3' | 124 |
|  | Primer R | 5'-ACATCACCCTCCCGAAACAG-3' |  |
| PIK3CB | Primer F | 5'-CTGCGACAGATGAGTGATGAAG-3' | 135 |
|  | Primer R | 5'-CCCTATCCTCCGATTACCAAG-3' |  |
| GDAP1 | Primer F | 5'-CTCATTCTGTACCATTGGACGC-3' | 125 |
|  | Primer R | 5'-CAAGGCTCATTGTGCTCACTC-3' |  |
| HIPK2 | Primer F | 5'-TCCAACTGGGACATGACTGG-3' | 145 |
|  | Primer R | 5'-CGATGGTCTGCTCGTAAGGTA-3' |  |
| G3BP1 | Primer F | 5'-CTGAAATCCAAGAGGAAAAGCC-3' | 142 |
|  | Primer R | 5'-GTCACAGATGCCCAAGAAAATG-3' |  |
| CD44 | Primer F | 5'-TGGGTTCATAGAAGGGCACG-3' | 106 |
|  | Primer R | 5'-ATACTGGGAGGTGTTGGATGTG-3' |  |
| NFKBIA | Primer F | 5'-CTCCATCCTGAAGGCTACCAA-3' | 102 |
|  | Primer R | 5'-GCACCCAAGGACACCAAAAG-3' |  |
| TGFB2 | Primer F | 5'-CCCACTTTCTACAGACCCTACTTC-3' | 141 |
|  | Primer R | 5'-AATCCGTTGTTCAGGCACTC-3' |  |
| CASP1 | Primer F | 5'-ATGCCTGTTCCTGTGATGTGG-3' | 102 |
|  | Primer R | 5'-AAGTCACTCTTTCAGTGGTGGG-3' |  |
| CCNA1 | Primer F | 5'-TGGAAAGAAAGCACTCCCTGAC-3' | 129 |
|  | Primer R | 5'-TCCTCAAATGCCATCCCCTC-3' |  |
| ATF3 | Primer F | 5'-AGGATTTTGCTAACCTGACGC-3' | 148 |
|  | Primer R | 5'-ACCTCGGCTTTTGTGATGG-3' |  |
| EZR | Primer F | 5'-AAGCTGGATAAGAAGGTGTCTGC-3' | 119 |
|  | Primer R | 5'-TGGGTGATGTCCTGGATGAG-3' |  |
| STC1 | Primer F | 5'-GCTCCACTTTCCAAAGGATG-3' | 100 |
|  | Primer R | 5'-TCAGTGATGGCTTCAGGGTT-3' |  |
| GAPDH | Primer F | 5'-TGACTTCAACAGCGACACCCA-3' | 121 |
|  | Primer R | 5'-CACCCTGTTGCTGTAGCCAAA-3' |  |

# Supplementary Figures

**Supplementary Figure 1. POLE2 level is negatively associated with poor prognosis in RCC**

(A) Representative immunohistochemical staining for POLE2 in RCC and adjacent normal tissues. (B) Kaplan‑Meier curves of the progression-free survival of RCC patients with high or low POLE2 expression.

**Supplementary Figure 2. POLE2 knockdown RCC cell lines are successfully constructed**

(A) A498 and ACHN cells were infected with shCtrl or shPOLE2 lentivirus for 72 h and then captured the fluorescent signal by fluorescence microscopy. (B-C) The knockdown efficiency of POLE2 in A498 and ACHN cells infected with shCtrl or shPOLE2 lentivirus was detected by (C) qRT-PCR or (D) western blot, GAPDH served as loading control. ***P*<0.01, ****P*<0.001.

**Supplementary Figure 3. Knockdown of POLE2 expression inhibits RCC cell** **proliferation and migration**

(A) The clone formation ability of A498 cells infected with shPOLE2#2 lentivirus was detected by clone formation assays. (B) The migration ability of A498 cells infected with shPOLE2#2 lentivirus was detected by Transwell assays. (C) The clone formation ability of A498 and ACHN cells infected with shPOLE2#2 lentivirus was detected by soft agar colony formation assays.
